# Marital status and its correlation with age, race, and gender in prognosis of tonsil squamous cell carcinomas

**DOI:** 10.1515/med-2022-0568

**Published:** 2022-11-11

**Authors:** Yujiao Li, Chaosu Hu

**Affiliations:** Department of Radiation Oncology, Fudan University Shanghai Cancer Center, Shanghai 200032, China; Department of Oncology, Shanghai Medical College, Shanghai, China; Department of Radiation Oncology, Fudan University Shanghai Cancer Center, 270 Dong An Road, Shanghai 200032, China

**Keywords:** tonsil squamous cell carcinomas, marital status, gender, prognosis

## Abstract

The objective of this study was to assess the impact of marital status on tonsil squamous cell carcinomas (TSCCs) prognosis and to analyze whether the impact is correlated with gender, age, and race. We examined the clinicopathological variables using Chi-squared tests and evaluated the association between survival and different variables using the methods of Kaplan–Meier. Univariate and multivariate analyses were performed to determine the effects of each variable on survival. A total of 10,720 patients were analyzed. The rate of being married was higher among Asian or Caucasian, and this rate decreased with higher tumor stage. While both married male and female survivors benefit from their marital status, we found a differential in cancer-specific survival based on gender, with males benefitting more than females (*p* < 0.05). The same results were found in overall survival. Subgroup analysis shows that the protective effect of marriage was consistent in all patients except for N3 groups (all, *p* < 0.05). While there are survival benefits for married patients with TSCCs, married/partnered males may benefit more than females. Age, race, and gender could affect the correlation between marital status and survival.

## 1 Introduction

Tonsil squamous cell carcinoma (TSCC) is one of the most common oropharyngeal neoplasm and the incidence rates of TSCC have significantly increased in recent decades. With the development of treatment strategies, the survival of patients with TSCC has been significantly improved. Up-front surgery followed by adjuvant therapy, if appropriate, or radiotherapy alone has been the principal treatment modality for early-stage TSCC and postoperative radiotherapy has been used for close or positive resection margins, T3–4 tumors, and neck node metastases [1–3].

Recent studies assessing the effect of marriage on outcomes showed that marriage was associated with better survival, and the protective effect of marriage might result from that the married people were associated with earlier stage and were more likely to receive recommended or aggressive treatment, which was known as “spousal surveillance” [4–9]. In addition, married patients were considered to have more emotional and financial support, which helps them to prolong their overall survival (OS) [10–12]. However, analysis of marital subgroup, which might reveal the potential mechanism generating the influence of marital status on prognosis, was not further analyzed.

Understanding the correlation between marital status and gender, race and age is important for developing tailored interventions aimed at improving socio-emotional support for patients. Therefore, we investigated the clinical correlates between marital status and survival and whether the association varied by gender, race, and age for TSCC using the Surveillance, Epidemiology, and End Results (SEER) database.

## 2 Materials and methods

### 2.1 Data sources

We obtained data from the current SEER database, which consists of 18 population-based cancer registries. This database collects and publishes cancer prevalence and survival data covering approximately 28% of the total population in the United States. SEER*Stat Version 8.3.4 (http://www.seer.cancer.gov/seerstat) from the National Cancer Institute was used to identify eligible patients in this study. We included patients diagnosed with microscopically confirmed TSCC between 1 January 2004 and 31 December 2014. We selected patients with only one primary malignancy in their lifetime. We excluded patients mainly because of the lack of pathology type of tumor, unknown marital status, unknown racial information, or unstaged tumors. A total of 10,720 TSCC patients were included.

### 2.2 Statistical analysis

Descriptive statistics were used to examine the baseline characteristics of the patients. The primary study outcomes were OS and cancer-specific survival (CSS). OS was defined as time to the date of death due to any cause or the date of last follow-up. CSS was defined as time from initial treatment to death due to cancer. Kaplan–Meier survival curves were compared using the log-rank test. Hazard analysis was conducted using the Cox proportional hazards model. SPSS software, version 22.0 (SPSS, Chicago, IL, USA) was used for additional data processing. *p*-Value of <0.05 was considered statistically significant for all tests.

### 2.3 Bias

The main limitation is the inherent bias that exists in retrospective studies.

## 3 Results

### 3.1 Clinical characteristics of all patients

Among the 10,720 patients, the median age was 58 years old (range [interquartile range]: 17–99 [52–65] years old). More than 80% patients (8,835/82.4%) were males. Among the cohort of the patients, 6,389 (59.6%), 3,739 (34.9%), and 592 (5.5%) patients were married/partnered, divorced/separated/single, and widowed, respectively. According to the 6th or 7th edition of UICC/AJCC Staging System, 1,687 (15.8%) and 9,033 (84.2%) were stage I–II and stage III–IV, respectively. The rate of being married was higher among Asian or Caucasian, and this rate decreased with higher tumor stage. Moreover, married/partnered patients received more surgeries. The clinicopathological features stratified by marital status at diagnosis are listed in Table 1.

**Table 1:** Demographic characteristics of TSCC patients who were stratified by marital status

Features	*n*		%	Married/partnered	Divorced/separated/single	Widowed	*p*	
				*n*	*n*	*n*		
Gender							0.000	
Male	8,835		82.4	5,489	3,064	282		
Female	1,885		17.6	900	675	310		
Age							0.000	
<58	5,165		48.2	3,127	1,964	74		
≥58	5,555		51.8	3,262	1,775	518		
Race							0.000	
Caucasian	9,385		87.5	5,794	3,097	494		
Asian	376		3.5	247	108	21		
African American	959		8.9	348	534	77		
Stage							0.000	
I	683		6.4	402	213	68		
II	1,004		9.4	606	321	77		
III	2,316		21.6	1,426	756	134		
IVA	5,392		50.3	3,319	1,846	227		
IVB	1,041		9.7	498	481	62		
IVC	284		2.6	138	122	24		
T classification							0.000	
T1	3,123		29.1	2,007	964	152		
T2	4,465		41.7	2,760	1,477	228		
T3	1,234		11.5	672	476	86		
T4a	1,208		11.3	623	502	83		
T4b	690		6.4	327	320	43		
N classification							0.000	
N0	2,327		21.7	1,362	765	200		
N1	2,443		22.8	1,488	811	144		
N2a	1,236		11.5	794	401	41		
N2b	3,302		30.8	2,011	1,150	141		
N2c	938		8.8	503	392	43		
N3	474		4.4	231	220	23		
Grade							0.000	
1	458		4.3	264	165	29		
2	4,547		42.4	2,591	1,683	273		
3	5,715		53.3	3,534	1,891	290		
Surgery therapy							0.000	
Yes	5,891		55.0	3,811	1,820	260		
No	4,829		45.0	2,578	1,919	332		
Tumor location							0.000	
Tonsillar fossa	1,555		14.5	876	567	112		
Tonsillar pillar	737		6.9	408	273	56		
Overlapping lesion of tonsil	121		1.1	57	60	4		
Tonsil, NOS	8,307		77.5	5,048	2,839	420		

Abbreviations: NOS, not otherwise specified.

### 3.2 Survival

The overall mean follow-up of all patients in the cohort was 34.0 months (range [interquartile range], 0–131 [13–68] months) and mortality rate was equal to 25 per 100,000 persons-years. In the univariate analysis, age, gender, marital status, grade, T category, N category, and surgery therapies to the primary tumor were significantly associated with OS and CSS (*p* < 0.01) (Table 2).

**Table 2:** Univariate analysis of CSS stratified by gender

Prognostic factor	Male				Female			
	*p-*Value	HR	Lower 95% CI	Higher 95% CI	*p-*Value	HR	Lower 95% CI	Higher 95% CI
Age	0.00				0.00			
<58		1 (Reference)				1 (Reference)		
≥58		1.54	1.46	1.63		1.52	1.37	1.69
Marital status	0.00				0.00			
Married/partnered		1 (Reference)				1 (Reference)		
Divorced/separated/single		2.14	1.92	2.39		1.16	0.89	1.51
Widowed		3.46	2.70	4.43		1.89	1.37	2.60
Race	0.00				0.01			
Caucasian		1 (Reference)				1 (Reference)		
Asian		1.11	0.83	1.49		0.78	0.40	1.51
African American		2.42	2.09	2.81		2.31	1.71	3.12
Grade	0.00				0.00			
1		1 (Reference)				1 (Reference)		
2		0.81	0.62	1.05		0.89	0.56	1.41
3		0.58	0.45	0.76		0.61	0.38	0.98
T classification	0.00				0.00			
T1		1 (Reference)				1 (Reference)		
T2		1.64	1.39	1.94		1.81	1.28	2.56
T3		2.59	2.12	3.16		4.48	2.92	6.88
T4a		4.87	4.07	5.83		5.16	3.56	7.48
T4b		5.91	4.86	7.19		6.77	4.51	10.17
N classification	0.00				0.00			
N0		1 (Reference)				1 (Reference)		
N1		1.10	0.92	1.31		0.95	0.69	1.31
N2a		0.51	0.40	0.66		0.74	0.47	1.19
N2b		1.06	0.90	1.25		0.89	0.63	1.24
N2c		2.44	2.02	2.95		2.12	1.45	3.08
N3		1.90	1.51	2.40		3.41	2.05	5.66
Surgery therapy	0.00	3.17	2.82	3.55	0.00	3.68	2.87	4.71

Abbreviations: CI, confidence interval; HR, hazard ratio; *p*-values were calculated using an adjusted Cox proportional hazards model.

**Figure 1:**
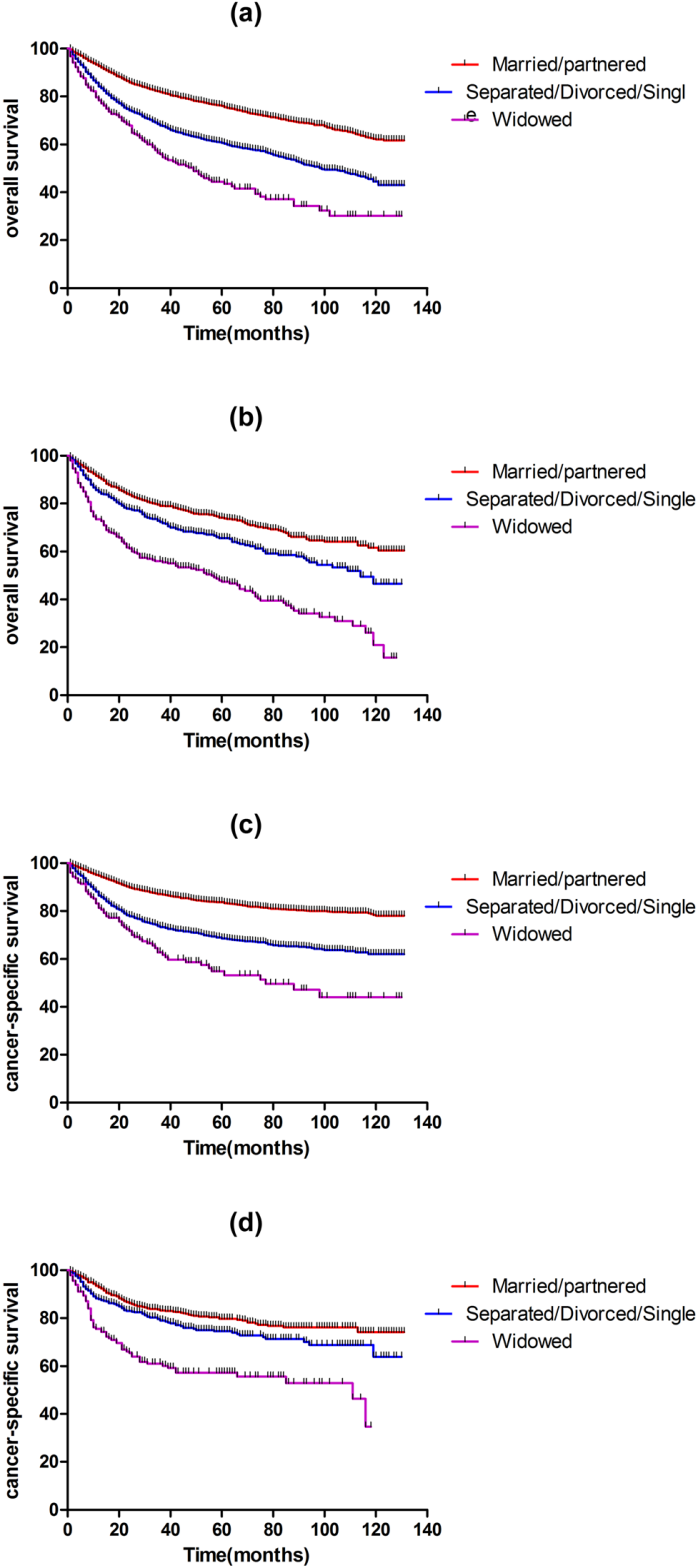
Kaplan–Meier analysis of OS and CSS in male and female TSCC patients: (a) OS in male TSCC patients (log rank *p* < 0.001), (b) OS in female TSCC patients (log rank *p* < 0.001), (c) CSS in male TSCC patients (log rank *p* < 0.001), and (d) CSS in female TSCC patients (log rank *p* < 0.001).

Kaplan–Meier analysis indicates that the married/partnered subgroup showed better OS and CSS than the unmarried groups, and the widowed patients showed worse prognosis for both genders (*p* < 0.001) (Figure 1).

These risk factors associated with all causes and CSS, identified in the univariate Cox proportional hazards regression model, were included in the multivariate analyses and age, marital status, grade, T category, N category, and surgery therapies to the primary tumor were all independent prognostic factors in the multivariable analysis. While marriage was associated with better survival for both married male and female, subgroups’ analysis showed a differential in CSS based on gender, with males benefitting more than females (*p* < 0.01) (Table 3). The same results were found in OS.

**Table 3:** Multivariable Cox proportional hazards analysis for CSS stratified by gender

Prognostic factor	Male				Female			
	*p-*Value	HR	Lower 95% CI	Higher 95% CI	*p-*Value	HR	Lower 95% CI	Higher 95% CI
Age	0.00				0.00			
<58		1 (Reference)				1 (Reference)		
≥58		1.67	1.50	1.87		1.59	1.23	2.06
Marital status	0.00				0.00			
Married/partnered		1 (Reference)				1 (Reference)		
Divorced/separated/single		1.73	1.55	1.94		1.16	0.89	1.51
Widowed		2.05	1.60	2.64		1.89	1.37	2.60
Race	0.00				0.01			
Caucasian		1 (Reference)				1 (Reference)		
Asian		1.12	0.83	1.50		0.82	0.42	1.60
African American		1.60	1.37	1.86		1.63	1.19	2.23
Grade	0.00				0.00			
1		1 (Reference)				1 (Reference)		
2		0.88	0.68	1.15		0.84	0.52	1.35
3		0.69	0.53	0.89		0.57	0.35	0.93
T classification	0.00				0.00			
T1		1 (Reference)				1 (Reference)		
T2		1.22	1.03	1.45		1.44	1.01	2.06
T3		1.53	1.24	1.89		2.77	1.77	4.34
T4a		2.77	2.29	3.35		3.36	2.26	4.98
T4b		3.20	2.61	3.94		4.08	2.63	6.34
N classification	0.00				0.17			
N0		1 (Reference)				1 (Reference)		
N1		1.24	1.04	1.48		1.14	0.83	1.58
N2a		0.71	0.55	0.91		0.98	0.61	1.58
N2b		1.09	0.92	1.29		0.83	0.59	1.17
N2c		1.64	1.35	1.99		1.48	1.00	2.19
N3		1.43	1.13	1.81		3.30	1.97	5.55
Surgery therapy	0.00	2.04	1.80	2.31	0.00	2.40	1.84	3.14

Abbreviations: CI, confidence interval; HR, hazard ratio; *p*-values were calculated using an adjusted Cox proportional hazards model.

### 3.3 Effects of marital status stratified by subgroups

To rule out the effects of these variables and further validate the effect of marital status on OS and CSS, we conducted the subgroup analysis based on these variables (Table 4). Remarkably, the protective effect of marriage was consistent in all patients except for N3 groups (all, *p* < 0.05).

**Table 4:** Effect of marital status on OS and CSS based on different subgroup variables

Subgroups	OS				CSS			
	*p-*Value	HR	Lower 95% CI	Higher 95% CI	*p-*Value	HR	Lower 95% CI	Higher 95% CI
Age								
<58	0.00	1.70	1.57	1.85	0.00	1.78	1.61	1.96
≥58	0.00	1.32	1.26	1.37	0.00	1.39	1.31	1.48
Race								
Caucasian	0.00	1.45	1.39	1.52	0.00	1.53	1.44	1.62
Asian	0.00	1.42	1.14	1.77	0.00	1.72	1.29	2.28
African American	0.00	1.36	1.23	1.50	0.00	1.43	1.25	1.64
Gender								
Male	0.00	1.53	1.46	1.61	0.00	1.66	1.55	1.77
Female	0.00	1.39	1.30	1.48	0.00	1.40	1.27	1.55
Grade								
1	0.00	1.32	1.13	1.55	0.00	1.53	1.23	1.92
2	0.00	1.46	1.38	1.54	0.00	1.56	1.45	1.68
3	0.00	1.48	1.40	1.57	0.00	1.56	1.44	1.68
T classification								
T1	0.00	1.45	1.32	1.58	0.00	1.38	1.18	1.62
T2	0.00	1.43	1.34	1.52	0.00	1.44	1.31	1.59
T3	0.00	1.43	1.29	1.58	0.00	1.58	1.38	1.81
T4a	0.00	1.48	1.36	1.62	0.00	1.63	1.46	1.82
T4b	0.00	1.36	1.21	1.52	0.00	1.34	1.17	1.53
N classification								
N0	0.00	1.43	1.33	1.53	0.00	1.51	1.35	1.70
N1	0.00	1.46	1.35	1.58	0.00	1.59	1.43	1.76
N2a	0.00	1.41	1.18	1.68	0.00	1.64	1.29	2.08
N2b	0.00	1.52	1.41	1.64	0.00	1.56	1.41	1.73
N2c	0.00	1.51	1.35	1.69	0.00	1.62	1.40	1.87
N3	0.04	1.21	1.01	1.46	0.06	1.22	0.99	1.50
Surgery therapy								
Yes	0.00	1.38	1.29	1.47	0.00	1.39	1.25	1.55
No	0.00	1.43	1.37	1.50	0.00	1.51	1.42	1.61
Stage								
I	0.00	1.42	1.24	1.62	0.05	1.35	1.00	1.81
II	0.00	1.46	1.30	1.64	0.00	1.44	1.17	1.78
III	0.00	1.40	1.29	1.53	0.00	1.56	1.38	1.76
IVA	0.00	1.55	1.46	1.64	0.00	1.68	1.55	1.82
IVB	0.00	1.36	1.23	1.50	0.00	1.37	1.21	1.54
IVC	0.00	1.31	1.12	1.52	0.00	1.34	1.11	1.61

Abbreviations: CI, confidence interval; HR, hazard ratio; *p*-values were calculated using an adjusted Cox proportional hazards model.

## 4 Discussion

We confirmed what previous studies had shown that marital status impacted on treatment outcome of TSCC patients in this study. Compared to other peers, the worse prognosis of widowed patients might have resulted from negative emotions and worse economic situation due to their unfavorable marital status. We further found that married/partnered males may benefit more than females, which relates to spousal influence on adoption of healthy behaviors, as well as the support to quit harmful risk factors associated with adverse outcomes [13]. Women tend to have a greater influence on their spouse’s health than men because women exert more effort to control their partners’ health habits [13,14]. Takagi et al. found that there was a spillover effect of the wife’s non-smoking only among men. However, husband’s non-smoking was not associated with female target’s cessation [15]. Generally speaking, the support received by male patients to adopt healthier lifestyles and have a positive outlook may explain their greater survival benefit from being married, which could partly explain the observed sex-based differences. The differential protective effect of marriage based on gender among TSCC patients is a novel finding, which is important for care planning and needs further exploration of the underlying specific mechanism behind this observation.

We then explored the effect of marital status on prognosis by different subgroups, such as age, race, stage, grade, and the surgery situation of patients. Difference of protective effect of marriage was found among variable subgroups except for N3 patients. Not only cancer, marriage also played a positive role in overall health. Kubzansky reported that marriage protected people from type 2 diabetes through favorable changes in lifestyle [16]. Another study of African Americans with heart failure proved that being married and living with family independently predict lower mortality and fewer readmissions [17]. Besides, a research of East Asian populations revealed marriage and marital satisfaction was of great importance in determining self-rated health [18]. These studies were compatible with our results that the greater impact of marital status on OS than CSS among variable subgroups.

As far as we know, this is the first SEER analysis assessing impact of age, race, and gender on the association between marital status and outcomes in TSCC. Several limitations were noted in this study. First, the SEER database does not include change of marital status after cancer diagnosis. Second, the lack of data on additional predictors of OS such as human papillomavirus infection, p16 status, performance status, comorbidities, tobacco smoking, alcohol consumption, betel nut, and mieng (a fermented ea-leaf) chewing, prevented us to adjust our analyses for these important factors. Finally, also due to the data limitations of the SEER database, positive surgical margins at final pathology were not able to be analyzed between marital status and CSS in this study.

## 5 Conclusion

Our results showed that while there are survival benefits for married/partnered patients with TSCC, married females may benefit more than males. Age, race, and gender could affect the correlation between marital status and survival.
